# A Case of Legg-Calvé-Perthes Disease due to Transient Synovitis of the Hip

**DOI:** 10.1155/2016/7426410

**Published:** 2016-07-11

**Authors:** Tadahiko Ohtsuru, Yasuaki Murata, Yuji Morita, Yutaro Munakata, Yoshiharu Kato

**Affiliations:** Department of Orthopedic Surgery, Tokyo Women's Medical University, 8-1 Kawada-cho, Shinjuku Ward, Tokyo 162-8666, Japan

## Abstract

Transient synovitis (TS) of the hip develops spontaneously in childhood; it usually has a good prognosis and is a self-limiting disease. However, its pathology is not well known. We describe a case of Legg-Calvé-Perthes disease (LCPD) that seemingly developed due to TS. Even if TS is diagnosed on the basis of the patient's medical history and imaging findings, physicians should consider the possibility of LCPD and perform a careful observation if joint effusion continues and/or a symptom does not improve within 4 weeks.

## 1. Introduction

Transient synovitis (TS) of the hip has a good prognosis, and it is a self-limiting disease. However, if the diagnosis of TS is delayed, it may progress to Legg-Calvé-Perthes disease (LCPD), which has a poor prognosis. The causal association between TS and LCPD is not well understood. We describe a case of LCPD that was seemingly due to TS and present a literature review.

## 2. Case Presentation

**Figure 1:**
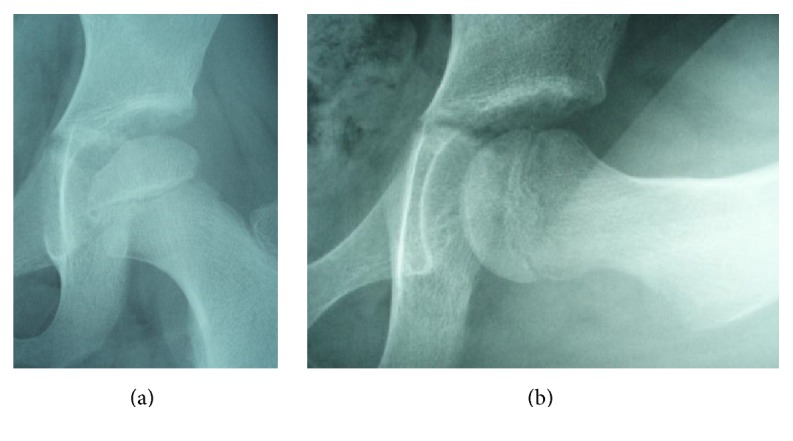
Plain radiographs of the hip joints at the initial visit. (a) Anteroposterior image. (b) Lateral image. No abnormalities were observed.

**Figure 2:**
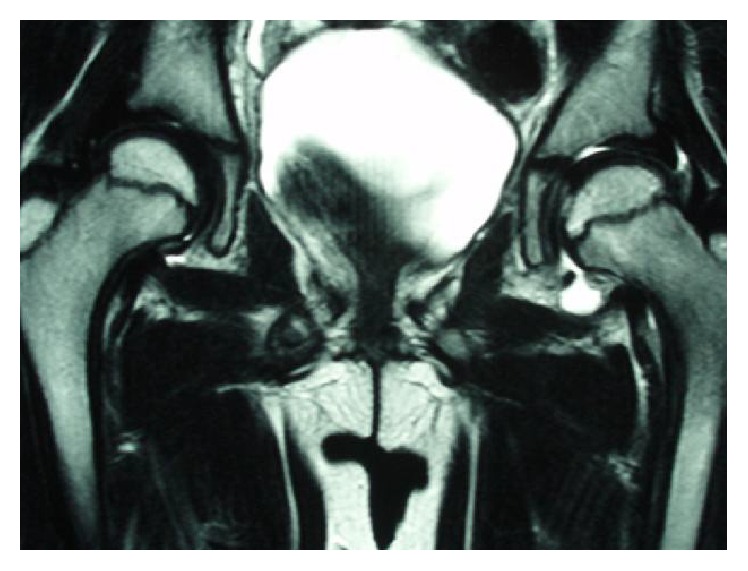
Coronal, T2-weighted magnetic resonance imaging scan at the initial visit. No abnormal findings, except left hip joint effusion, were observed.

**Figure 3:**
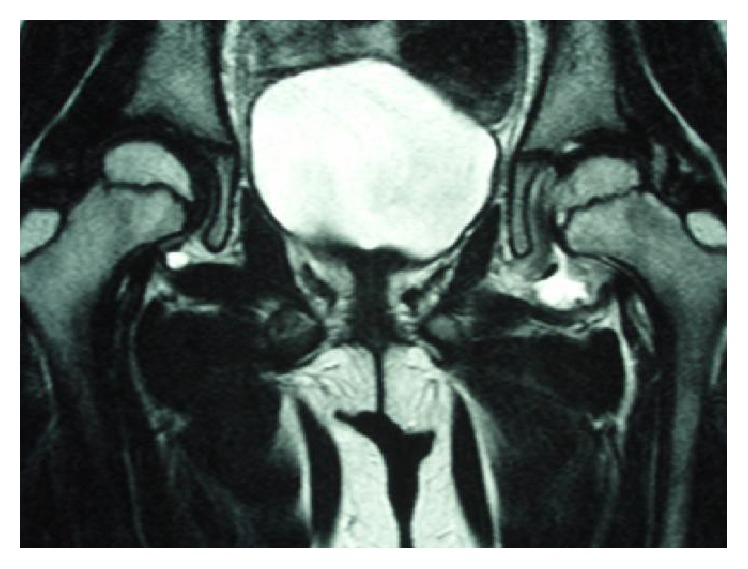
Coronal, T2-weighted magnetic resonance imaging scan of the hip joints 2 months after the initial visit. A linear low-signal-intensity region was found at the left femoral capital epiphysis.

**Figure 4:**
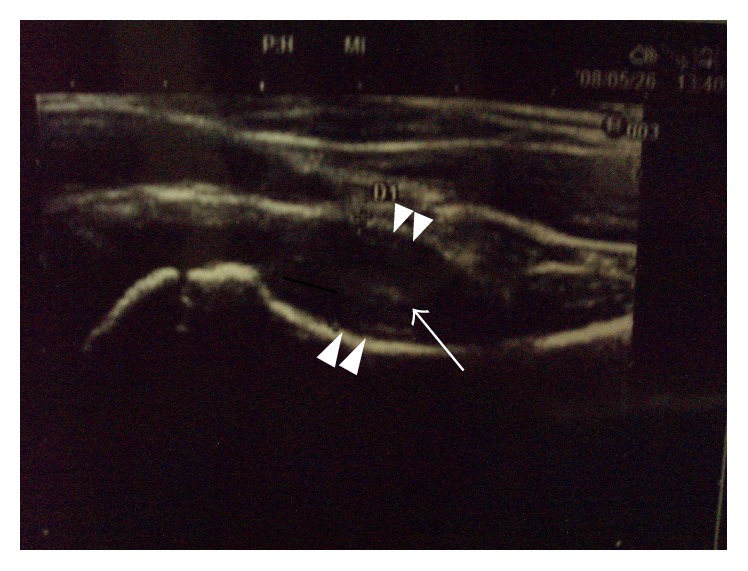
Ultrasonic joint space (distance from the anterior cortex of the femoral neck to the front of the capsule [the width of the joint space]) 2 months after the initial visit. It was 11.6 mm for the left hip (arrowheads). The intracapsular space showed echoic findings (arrow).

**Figure 5:**
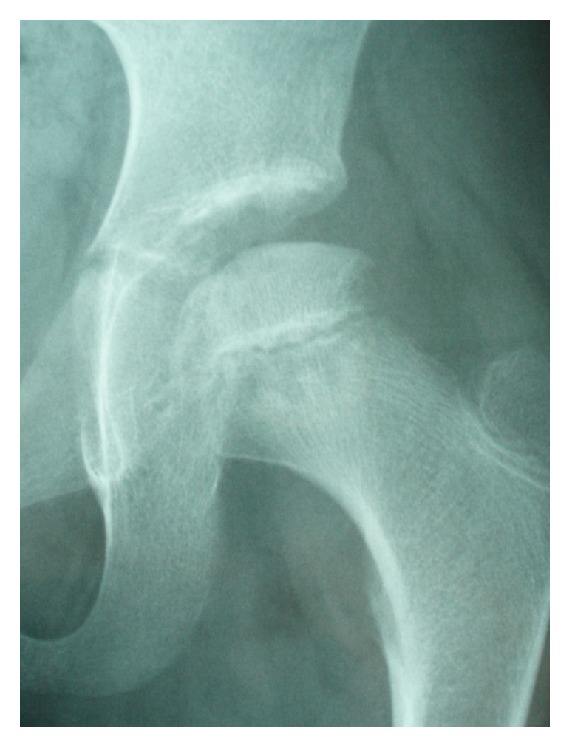
Plain radiograph of the hip joint 3 months after the initial visit. Bone resorption was observed at the left femoral capital epiphysis.

**Figure 6:**
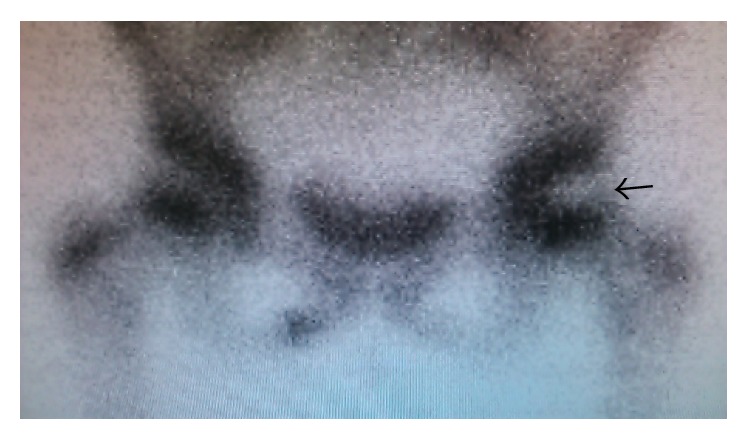
Bone scintigraphy findings. A cold-in-hot pattern is shown at the left femoral capital epiphysis (arrow).

**Figure 7:**
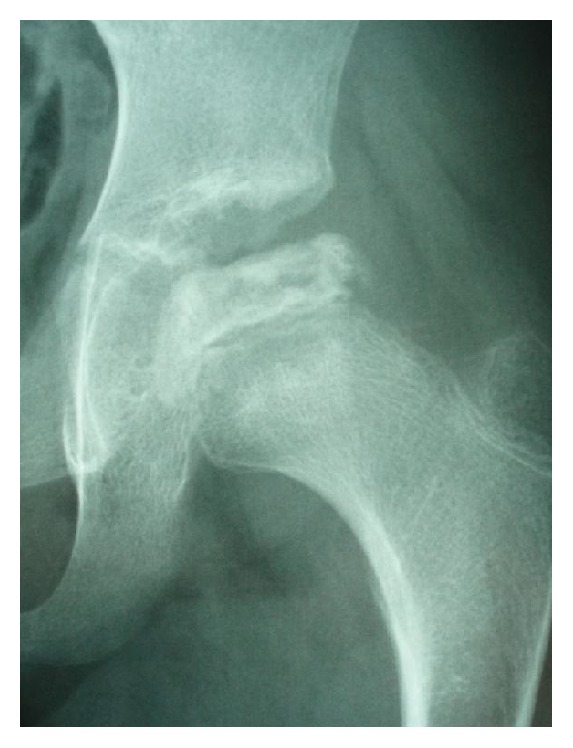
Plain radiograph of the left hip joint 8 months after the initial visit. We categorized the disease as Catterall group II and Herring group B.

**Figure 8:**
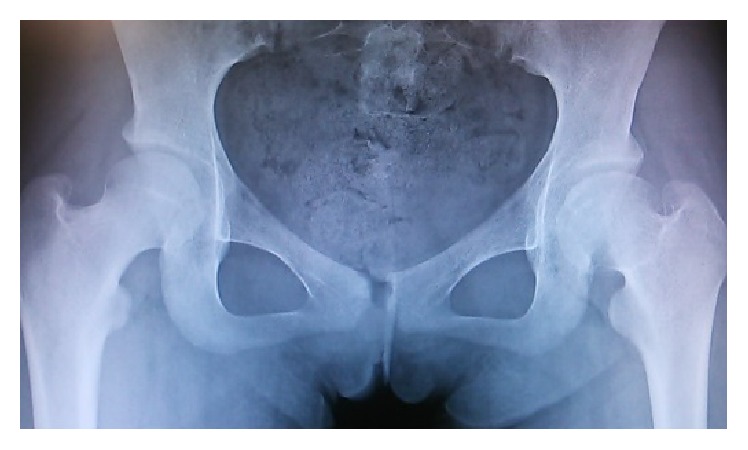
Plain radiograph of the hip joints 7 years after the initial visit. Good remodeling of the necrotic area at the left femoral capital epiphysis was observed.

A 7-year-old girl complained of pain in her left thigh, and she was limping. She had no history of trauma, disease, or surgery. She had left thigh pain for 3 weeks before visiting the outpatient clinic. The ranges of left hip motion were as follows: flexion, 130°; abduction, 30°; extension, 10°; internal rotation, 45°; and external rotation, 10°. The circumferences of the right and left thighs were 29.5 cm and 28.5 cm, respectively. Plain radiographs obtained at the initial visit showed no abnormalities (Figure 1). T2-weighted magnetic resonance imaging (MRI) scan of the hip joints showed no abnormal findings, except left hip joint effusion (Figure 2). TS was diagnosed on the basis of the patient's medical history and imaging findings, and we continued follow-up. She was on bedrest for 4 weeks, and we instructed her to avoid weight bearing during this time. However, her left thigh pain and limping did not improve. The ultrasonic joint spaces (distance from the anterior cortex of the femoral neck to the front of the capsule [the width of the joint space]) measured 6.0 mm on the right side and 8.7 mm on the left side at 4 weeks after the initial visit. Joint effusion was diagnosed on the basis of anechoic findings of the left hip joint space. T1-weighted and T2-weighted MRI scans of the hip joints showed a linear low-signal-intensity region at the left femoral capital epiphysis 2 months after the initial visit (Figure 3). Although no abnormalities were found, follow-up continued. The ultrasonic joint space measured 11.6 mm on the left side 2 months after the initial visit (Figure 4). Synovitis was diagnosed on the basis of echoic findings of the left hip joint space. A culture of the joint aspirate showed negative results. The laboratory data showed no abnormalities. The ultrasonic joint space measured 12.3 mm on the left side 3 months after the initial visit. Hence, we determined that synovitis progressed. Plain radiograph showed bone resorption at the left femoral capital epiphysis 3 months after the initial visit (Figure 5). Bone scintigraphy findings showed a cold-in-hot pattern at the left femoral capital epiphysis (Figure 6). We diagnosed the patient as having LCPD on the basis of these imaging findings. We instructed her to wear a hip abduction and non-weight-bearing brace. We categorized the disease as Catterall group II and Herring group B according to the findings from plain radiographs 8 months after the initial visit (Figure 7). Plain radiographs showed a continuous subchondral bone 15 months after the initial visit. We removed her brace and continued follow-up. Plain radiographs showed good remodeling of the necrotic area at the left femoral capital epiphysis 7 years after the initial visit. She did not have a lower limb discrepancy, left thigh pain, or limping (Figure 8).

## 3. Discussion

Hip diseases in school-age children are categorized as a good-prognosis or poor-prognosis disease. TS is a self-limiting, good-prognosis disease, whereas LCPD, slipped capital femoral epiphysis, and septic arthritis of the hip are poor-prognosis diseases if the diagnosis is delayed. Although most hip diseases in school-age children can be easily diagnosed, some cases are difficult to diagnose. In addition, the association between TS and LCPD is still unclear.

Plain radiographs of the present patient showed no abnormalities at the time of pain onset. T2-weighted MRI scans and ultrasonogram of the hip joints showed no abnormal findings, except left hip joint effusion. Thus, we diagnosed the present patient as having TS. However, her left thigh pain and limping did not improve. Although T1-weighted and T2-weighted MRI scans of the hip joints showed a linear low-signal-intensity region at the left femoral capital epiphysis 2 months after the initial visit, we determined that these findings did not indicate anything significant. Finally, LCPD was diagnosed on the basis of the findings from plain radiographs and bone scintigraphy 3 months after the initial visit.

In 1984, Wilson et al. first reported the usefulness of ultrasonography for diagnosing coxarthrosis in childhood [1]. Zieger et al. reported that if the intracapsular intensity on an ultrasonogram was anechoic, it indicated effusion or fresh blood, and if the intracapsular intensity on an ultrasonogram was echoic, it indicated septic arthritis, old blood, synovitis, or a tumor [2]. The period between the onset and disappearance of symptoms was about 2 weeks in cases of TS [1, 3, 4]. Hattori et al. reported on discriminating between early LCPD and TS by measuring the ultrasonic joint space [5]. They concluded that although the ultrasonic joint space normalized within 2 weeks in cases of TS, it did not improve for more than 4 weeks in cases of LCPD. The normal values of the ultrasonic joint space in children reportedly range from 5 to 6 mm [5, 6]. Thus, the ultrasonic joint spaces on both sides of the hip must be compared, because the spaces may be large in a healthy patient [7].

**Figure 9:**
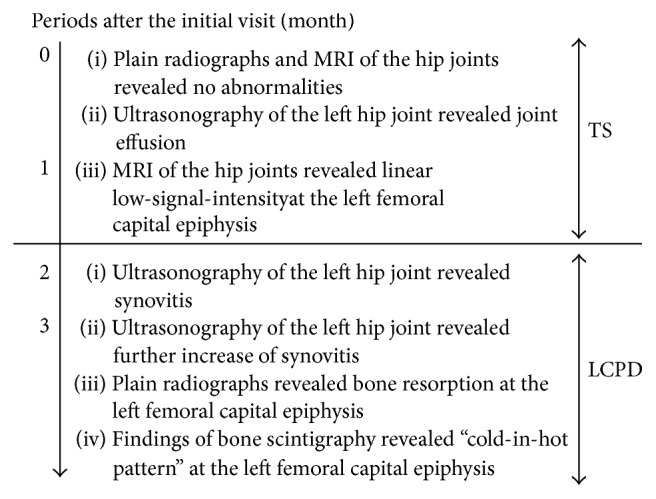
Flowchart of the imaging findings in the present case. MRI: magnetic resonance imaging, TS: transient synovitis, and LCPD: Legg-Calvé-Perthes disease.

Considering these previous reports, a patient with symptoms that do not improve within more than 4 weeks should be considered as having LCPD. As the present patient had a typical clinical course and imaging findings of TS for the initial 4 weeks and those of LCPD for more than 4 weeks later (Figure 9), we suspected that there was an association between these two diseases.

The causal association between TS and LCPD is not well understood. Bickerstaff et al. [4], Terjesen and Østhus [8], and Landin et al. [9] reported cases that have likely developed LCPD due to TS [4, 8, 9]. In contrast, Kallio et al. did not support the association between TS and LCPD [3]. Futami et al. described the case of a 10-year-old boy who developed right LCPD during follow-up for left LCPD [10]; MRI was performed incidentally 5 weeks before right LCPD developed. T1-weighted MRI scan showed a low-intensity band region at the femoral capital epiphysis, and T2-weighted MRI scan showed a high-intensity band region at the femoral capital epiphysis. They reported that these findings indicated bone marrow edema. Similarly, MRI was performed before necrosis developed in the present case, and T1-weighted and T2-weighted MRI scans showed a linear low-signal-intensity region at the femoral capital epiphysis (Figure 3). As the findings of this region coincided with the medial margin of necrosis, which developed later, we considered that this finding indicated a small portion of the necrotic region just after it began to develop.

Trueta reported that LCPD was caused by vascular insufficiency of the lateral epiphyseal artery (the branch of the medial circumflex femoral artery) [11]. We considered that avascular necrosis of the femoral capital epiphysis was caused by the increase in intracapsular pressure due to prolonged effusion and synovitis, as reported by Wlngstrand et al. [12].

In conclusion, we described a case of LCPD seemingly due to TS. Even if TS is diagnosed on the basis of a patient's medical history and imaging findings, physicians should consider LCPD and perform careful observation if joint effusion continues and/or a symptom does not improve within 4 weeks.
